# Quality of Life (QoL) Is Reduced in Those with Severe COVID-19 Disease, Post-Acute Sequelae of COVID-19, and Hospitalization in United States Adults from Northern Colorado

**DOI:** 10.3390/ijerph182111048

**Published:** 2021-10-21

**Authors:** Kim McFann, Bridget A. Baxter, Stephanie M. LaVergne, Sophia Stromberg, Kailey Berry, Madison Tipton, Jared Haberman, Jeremy Ladd, Tracy L. Webb, Julie A. Dunn, Elizabeth P. Ryan

**Affiliations:** 1 Medical Center of the Rockies, University of Colorado Health, Loveland, CO 80538, USA; mtipton@rams.colostate.edu (M.T.); julie.dunn@uchealth.org (J.A.D.); 2 Department of Environmental Radiological and Health Sciences, College of Veterinary Medicine and Biomedical Sciences, Colorado State University, Fort Collins, CO 80523, USA; bridget.baxter@colostate.edu (B.A.B.); stephanie.lavergne@colostate.edu (S.M.L.); sophia.stromberg@rams.colostate.edu (S.S.); kailey.berry@colostate.edu (K.B.); jaredh17@rams.colostat.edu (J.H.); jeremy.ladd@rams.colostate.edu (J.L.); 3 Department of Clinical Sciences, College of Veterinary Medicine and Biomedical Sciences, Colorado State University, Fort Collins, CO 80523, USA; tracy.webb@colostate.edu

**Keywords:** COVID-19, quality of life, SF-36, post-acute sequelae of COVID-19 (PASC), hospitalization, COVID-19 severity

## Abstract

The longitudinal quality of life (QoL) of COVID-19 survivors, especially those with post-acute sequelae (PASC) is not well described. We evaluated QoL in our COVID-19 survivor cohort over 6 months using the RAND SF-36 survey. From July 2020–March 2021 we enrolled 110 adults from the United States with a positive SARS-CoV-2 nasopharyngeal polymerase chain reaction (PCR) into the Northern Colorado Coronavirus Biobank (NoCo-COBIO). Demographic data and symptom surveillance were collected from 62 adults. In total, 42% were hospitalized, and 58% were non-hospitalized. The Rand SF-36 consists of 36 questions and 8 scales, and questions are scored 0–100. A lower-scale score indicates a lower QoL. In conclusion, hospitalization, PASC, and disease severity were associated with significantly lower scores on the RAND SF-36 in Physical Functioning, Role Limitation due to Physical Health, Energy/Fatigue, Social Functioning, and General Health. Long-term monitoring of COVID-19 survivors is needed to fully understand the impact of the disease on QoL and could have implications for interventions to alleviate suffering during recovery.

## 1. Introduction

The impact of the SARS-CoV-2 infection on physical and mental health, as well as social and emotional well-being, merits standardized evaluation and international research attention. Many COVID-19 survivors suffer from persistent dyspnea, fatigue, insomnia, and anxiety, amongst a range of other symptoms [1,2,3,4,5,6,7,8,9]. COVID-19 survivors also report low quality of life (QoL) 1–3 months after infection, in addition to significant impairment in physical and psychological functioning [10,11,12]. Physical health is closely related to QoL, and patients with measurable decreases in pulmonary function following COVID-19-related pneumonia also reported lower QoL [13]. Moreover, hospitalized patients who survived COVID-19 reported a decrease in short-term QoL [14,15,16]. The longitudinal QoL in survivors of COVID-19, especially those with post-acute sequelae of COVID-19 (PASC) lasting 6 months or more, needs further attention. The persistence of physical and mental COVID-19 symptoms have further challenged the concept of QoL [3] and likely contribute to the loss of QoL. We aimed to evaluate for differences in QoL in those with and without PASC. For this purpose, we chose to administer the RAND version of the SF-36. The Rand SF-36 consists of 36 questions and eight scales: Physical Functioning, Role Limitations due to Physical Health, Role Limitations due to Emotional Problems, Energy/Fatigue, Emotional Well-being, Social Functioning, Pain, and General Health. Questions are scored 0–100, and the higher the score, the better the level of functioning. This instrument has been validated for use in health related QoL, such as chronic headaches, Amyotrophic Lateral Sclerosis, traumatic brain injury, and stroke. In addition, the time to administer is minimal (approximately 10–15 min) compared to the WHOQOL-100, which is an internationally used QOL instrument that takes anywhere from 30–90 min depending on the literacy level of participants. 

Colorado State University (CSU), in conjunction with University of Colorado Health (UC Health), established the Northern Colorado Coronavirus Biorepository (NoCo-COBIO), an observational, longitudinal cohort of individuals infected with SARS-CoV-2 [17]. One objective of the biorepository is to collect information on the QoL of COVID-19 survivors using the RAND version of the SF-36 in the convalescent phase of the disease. The NoCo-COBIO includes multiple visits during the first 180 days from the initial polymerase chain reaction (PCR)+ diagnosis and involves a one year follow up. We hypothesized that participants with severe and moderate disease would have a decreased QoL compared to those with mild disease. We also hypothesized that those who experienced hospitalization and post-acute sequelae of COVID-19 (PASC) would have decreased QoL scores compared to those who were not hospitalized and did not develop PASC.

## 2. Materials and Methods

### 2.1. Study Design 

An observational, longitudinal cohort design was used to recruit COVID-19 survivors to participate through the University of Colorado Health (UC Health) and Colorado State University (CSU) networks, as previously described [17]. Inclusion criteria for this study were a positive SARS-CoV-2 PCR test and ≥18 years. Pregnancy was an exclusion criterion. Each participant consented to undergo clinic visits at 4 different time points: at enrollment, and 1 month, 3 months, and 6 months after enrollment. Pre-existing conditions were collected from hospital electronic records and self-reported at the clinic visits. Body mass index (BMI) was calculated by self-reported height and weight for the non-hospitalized participants (*n* = 36), and hospital records were used for height and weight from hospitalized participants (*n* = 26). The National Institute of Health’s body mass calculator was accessed on 2 January 2021, (https://www.nhlbi.nih.gov/health/educational/lose_wt/BMI/bmicalc.htm) to calculate BMI. The RAND SF-36 was administered at least 15 days after infection and will be re-administered at 6 months and one year of follow-up for this biorepository cohort. All enrolled participants provided written informed consent. The study met the Helsinki Declaration guidelines. This research study, from the Northern Colorado Coronavirus Biobank (NoCo-COBIO), has been approved by CSU’s Research Integrity and Compliance Review Office Internal Review Board (IRB; protocol ID 20-10063H), as well as UC Health’s IRB (Colorado Multiple IRB 20-6043), and is registered with ClinicalTrials.gov (NCT05603677). The cohort was examined for the relationship of disease severity, hospitalization, and PASC with QoL using the RAND SF-36 survey [18]. The complete study design is illustrated in Supplemental Figure S1. 

### 2.2. Participants

Participants were categorized as having mild, moderate, or severe disease based on the Yale Impact Score which used oxygen requirements during the acute phase of COVID-19: no oxygen use was categorized as mild, a 1–5 L/min oxygen requirement was considered moderate, and a greater than 5 L/min oxygen requirement was documented as severe disease [19]. Demographic data and hospitalization status were obtained from participants at their clinic visits. Symptom surveillance was administered to evaluate new or persistent symptoms since the initial COVID-19 diagnosis at each follow-up visit. PASC was defined as having at least one of the following symptoms reported during the longitudinal surveillance period: fatigue, dyspnea, joint pain, chest pain, or cognitive dysfunction, at any or all of the follow-up visits [17]. Cognitive dysfunction was defined as absent-mindedness, forgetfulness, confusion, or problems with concentration. All data were de-identified and password-protected for inclusion in the database and prior to analysis. 

### 2.3. Instrument 

The Rand SF-36 consists of 36 questions and 8 scales measuring QoL [18]. The scales are: Physical Functioning, Role Limitations due to Physical Health, Role Limitations due to Emotional Problems, Energy/Fatigue, Emotional Well-being, Social Functioning, Pain, and General Health. All questions are scored 0–100, and a combination of questions make up the scales. The higher the score on a question, and ultimately a scale, the better the level of functioning [10,20]. Considerable evidence was found for the reliability of the SF-36 (Cronbach’s alpha greater than 0.85, and reliability coefficient greater than 0.75 for all dimensions except social functioning). Construct validity was confirmed by distinguishing between groups with expected health differences. To demonstrate validity, the SF-36 was able to detect low levels of ill health in participants who had scored 0 (good health) on the Nottingham health profile, the reverse scoring of the SF-36 [21,22]. For this study, we created an electronic version of the RAND SF-36 which was completed in person with the study team and which automatically calculated scores for the 8 scales, saving time and ensuring accuracy. Either Collaborative Institutional Training Initiate (CITI)-trained biomedical students, the study coordinator, team MD, and/or the principal investigators verbally administered the Rand SF-36 QoL questionnaire privately to the participants at the end of the study visits. 

### 2.4. Statistical Analysis

Demographic data were presented as Mean ± SD or Frequency and percent (%). Independent T-tests or ANOVA (with a Tukey–Kramer *p*-value adjustment to determine which groups differed) were used to test the differences among characteristics of COVID-19 participants on all 8 scales of the Rand Version of the SF-36. ANCOVA was performed using body mass index (BMI) and age as covariates. All analyses were performed using SAS 9.4 (Cary, NC, USA). *p* < 0.05 was considered significant without adjustment for multiple tests. 

## 3. Results

### 3.1. Participant Characteristics 

Data were collected on 62 participants from the NoCo-COBIO cohort with a mean age of 51.8 ± 16.6 years (Table 1). Most participants were non-Hispanic (53; 85%) and female (38; 61%) with no pre-existing medical conditions. Many participants were obese (27; 44%) or overweight (15; 24%), with a mean BMI of 30.3 ± 7.9. In all, 58% of participants had mild COVID-19 at the time of acute illness, 19% had moderate disease, and 23% suffered severe disease. A total of 26 (42%) participants were hospitalized for COVID-19, and 32 (51.6%) had PASC (Table 1). 

### 3.2. Quality of Life

In total, 62 participants underwent the RAND SF-36 QoL surveillance. All participants were at least 15 days post-PCR+ test results at time of questionnaire. Mean day post PCR+ was 125.3 ± 71.8 days. 

Participants requiring hospitalization were compared to those who did not, and we found that hospitalized participants scored significantly on the following scales: Physical Functioning (*p* < 0.0001), Role Limitations due to Physical Health, Role limitations due to Emotional Problems, Energy/Fatigue, Social Functioning, and General Health (*p* < 0.05) (Table 2, Supplemental Figure S2). 

All hospitalized participants suffered either moderate or severe disease, as shown in Table 3. As expected, those with moderate and severe disease scored significantly lower than participants with mild disease on the same five scales (Physical Functioning, Role Limitation due to Physical Health, Energy/Fatigue, Social Functioning, and General Health) Table 3.

SF-36 scores were compared in participants with and without PASC, and those with PASC had significantly lower scores in Physical Functioning, Role Limitation due to Physical Health, Role Limitation due to Emotional Problems, Energy/Fatigue, Social Functioning, Pain, and General Health (Supplemental Figure S3 and Table 4). 

SF-36 scores were compared between those with and without pre-existing medical conditions (Table 5). Participants with chronic conditions had significantly lower scores in the following: Physical Functioning, Role Limitations due to Physical, Energy/Fatigue, and General Health (Table 5). 

Additionally, SF-36 scores were evaluated for differences due to participant sex, ethnicity, and days post-SARS-CoV-2 PCR positive test. Females had a significantly lower Pain scale score than males (Supplemental Table S1). There was no difference between Hispanic and non-Hispanic participants in any of the scales (Supplemental Table S2). No significant differences were found in any of the eight scales between those who were 15–44-days post PCR+ and those who were 175+ days post PCR+ (Supplemental Table S3).

Lastly, SF-36 scores were compared across body weight categories based on CDC definitions for BMI (≤19.9 = underweight, 20–24.9 = normal, 25–29.9 = overweight, >30 = obese). Obese individuals had reduced measures for Physical, Role Limitation due to Physical Health, Energy/Fatigue, and General Health compared to those who were normal weight or underweight (Table 3). ANCOVA was performed with BMI and age as continuous covariates in models, testing PASC, hospitalization, and disease severity as the independent variables and with the 8 SF-36 scales as the outcome variables. BMI and age were not significantly related to PASC, hospitalization, and chronic conditions (Supplemental Table S4). 

## 4. Discussion

This study demonstrates that severe and moderate COVID-19 has a greater effect on QoL when compared to those with mild disease. Notably, most of the moderate and severe disease patients in this cohort were also hospitalized. We found significantly decreased scores in Physical Function, Role Limitations Due to Physical Health, Energy/Fatigue, Pain, Social Functioning, and General Health scales. Pre-existing medical conditions, PASC, and BMI categories also resulted in lower QoL scores. While those who were obese had lower scores on most SF-36 scales, BMI as a continuous covariate was not significantly related to any of the SF-36 scale scores, nor was age. 

Physical Functioning and Energy/Fatigue scales had the lowest scores on the RAND SF-36 QoL survey [23,24,25,26,27]. These results are consistent with previous studies, showing that 50–75% of non-hospitalized patients reported fatigue after COVID-19 [27]. Previous studies have shown that QoL is diminished at 1–3 months post-SARS-CoV-2 infection [20,22,28]; however, these participants from the U.S. still had lower QoL 6-months after PCR+ diagnosis. Although some studies have found an association between obesity and severe COVID-19 disease, the BMI dropped out of the models when included as a continuous covariate with the other significant variables in this study [23]. 

Considerable attention has been raised by COVID-19 survivors who have lingering symptoms for months after the infection. PASC affects patients across the spectrum of disease severity [3,27,28,29,30,31,32]. This study validates the significant impact of PASC on survivors’ QoL. Further research into the mechanisms behind PASC, early identification of those who will develop PASC, and methods to prevent, treat, or support those who develop PASC is warranted. Interestingly, despite the physical impact of COVID-19, Emotional Well Being on the SF-36 was not significantly impacted in any of the patient groups, even those with PASC; however, the scale for Role limitations due to Emotional Problems was impacted in those who were hospitalized or experienced PASC. Previously, chronic conditions, such as asthma, have been associated with severity of COVID-19 and correlated with decreases in all SF-36 scores [33]. Doll et al. reported that obesity has a higher impact on physical rather than emotional well-being measures, and proposed that assessments of emotional well-being can be confounded by the presence of chronic comorbidities [23]. Although Emotional Well Being was not impacted in this sample, those with PASC had significantly lower scores for Role Limitations due to Emotional Problems. Continued investigation of patients with PASC is warranted. 

This study emphasizes that COVID-19 affects long-term patient health, an awareness of which can assist clinicians in identifying those who may be at risk for diminished QoL, such as the hospitalization of patients, presence of PASC, and those having underlying chronic conditions including obesity. Accordingly, the medical and scientific community can work together with these patients to improve their QoL over the long-term. 

Some limitations are present in this study. First, our sample was limited to participants residing in Northern Colorado within the U.S. Second, we did not have baseline SF-36 QoL scores for these adults prior to acute COVID-19 infection that could be compared to scores during the convalescence stages of the disease. Third, the assessment of PASC related to QoL may have been influenced by the definition of PASC used. The longitudinal symptom surveillance included ongoing fatigue in response to COVID-19, and the SF-36 instrument captures fatigue as one of the questions for the Energy/Fatigue scale. Fourth, physical isolation has occurred more frequently during the COVID-19 pandemic, and is associated with stress, depression, unhealthy diet, and reduced physical activity in other countries which may have affected our results [28]. Finally, there may have been a potential bias due to participants responding to a clinician asking questions, compared to other study personnel.

## 5. Conclusions

We found that QoL in COVID-19 survivors is impacted by hospitalization, disease severity, PASC, and possibly obesity. These findings are important for clinicians across medical specialties and public health practitioners to assist COVID-19 survivors to regain QoL and prior functional status. The future of COVID-19 survivors, especially those with persistent symptoms and PASC remains unclear. As we gain more understanding of the effects of COVID-19, we can seek interventions to prevent and approach obstacles in survivors. The results of this study strongly support the continued longitudinal collection and assessment of QoL and symptom surveillance from COVID-19 survivors to help address concerns related to the identification of reduced QoL. Targeted approaches for the prevention, control, and treatment of PASC is needed to improve QoL outcomes globally. 

## Figures and Tables

**Table 1:** Demographics of COVID-19 adult participants that completed the Rand SF-36.

Characteristics	Mean ± SD Frequency (%)
Age	50.8 ± 16.6
BMI *	30.3 ± 7.9
Normal or Underweight^#^	20 (32.3%)
Overweight	15 (24.2%)
Obese	27(43.6%)
15–44 Days Post PCR+	11 (18.0%)
45–89 Days Post PCR+	16 (26.2%)
90–175 Days Post PCR+	17 (27.9%)
175+ Days Post PCR+	17 (27.9%)
Mean Days Post PCR+	125.3 ± 71.8
Male	24 (38.7%)
Female	38 (61.3%)
Hispanic	9 (14.5%)
Non-Hispanic	53 (85.5%)
Hospitalized	26 (42%)
Non-Hospitalized	36 (58%)
Disease Severity	
Mild	36 (58.1%)
Moderate	12 (19.4%)
Severe	14 (22.6%)
Post-Acute Sequelae COVID-19 (PASC)	32 (51.6%)
No PASC	30 (48.4%)
Pre-existing Medical Conditions	28 (45.2%)
No Pre-Existing Conditions	34 (54.8%)
Pre-existing Conditions	
Myocardial Infarction	1 (1.6%)
CVA	1 (1.6%)
COPD	5 (8%)
Connective Tissue Disease	1 (1.6%)
Liver Disease	2 (3%)
DM	11 (18%)
Lymphoma	2 (3%)
HTN	19 (31%)
Asthma	11 (18%)

Note: Disease severity was determined by oxygen use in accordance with the Yale Impact Score: mild (no oxygen required), moderate (1–5 L oxygen use), severe (<5 L oxygen use). Pre-existing conditions were collected from hospital electronic records and self-reported at clinic visits: CVA cerebrovascular accident, COPD chronic obstructive pulmonary disease, DM diabetes mellitus, HTN high blood pressure. Values are presented as mean ± standard deviation or frequency (percent). * BMI = Body Mass Index, PCR = polymerase chain reaction, PASC = post-acute sequelae of COVID-19; 3 participants were mildly underweight (BMIs = 18.8, 19.4, 19.9).

**Table 2:** Comparison of SF-36 scores in adults hospitalized for COVID-19.

SF-36 Scales	Hospitalized (N = 26)	Non-Hospitalized (N = 36)	*p*-Value
Physical Functioning	53.0 ± 31.5	90.2 ± 17.2	<0.0001
Role Limitations due to Physical Health	36.5 ± 37.6	73.6 ± 36.5	<0.001
Emotional Well Being	73.5 ± 24.5	74.5 ± 17.0	0.86
Role limitations due to Emotional Problems	59.0 ± 42.5	77.7 ± 30.9	<0.05
Energy/Fatigue	39.8 ± 25.9	56.8 ± 25.1	<0.05
Pain	71.8 ± 30.8	76.6 ± 25.5	0.50
Social Functioning	67.8 ± 27.2	84.8 ± 21.2	<0.01
General Health	56.7 ± 21.0	72.5 ± 24.2	<0.01

Values are presented mean ± standard deviation.

**Table 3:** SF-36 scores according to COVID-19 disease severity.

SF-36 Scales	Mild N = 36	Moderate N = 12	Severe N = 15	*p*-Value
Physical Functioning	90.2 ± 17.2	49.2 ± 36 *	56.3 ± 28.1 *	<0.0001
Role Limitations due to Physical Health	73.6 ± 36.5	31.3 ± 35.6 *	41.7 ±39.62 *	<0.01
Emotional Well Being	74.5 ± 17.0	69.7 ± 27.4	76.9 ± 22.3	0.66
Role limitations due to Emotional Problems	77.7 ± 30.9	66.7 ± 40.2	52.3 ± 44.7	0.09
Energy/Fatigue	56.8 ± 25.1	30.8 ± 23.4 *	47.5 ± 26.2	<0.05
Pain	76.6 ± 25.5	66.5 ± 3.13	6.45 ± 29.2	0.52
Social Functioning	84.8 ± 21.2	67.7 ± 27.4	67.9 ± 28.0	<0.05
General Health	72.5 ± 24.2	52.1 ± 22.1 *	60.7 ± 20.0	<0.05

Note * *p* < 0.05 compared to Mild. The Omnibus test for Social Functioning was significant, but there were no significant differences among the groups when Tukey’s post-hoc test was performed, which adjusts for multiple comparisons. Values presented are mean ± standard deviation.

**Table 4:** Comparison of SF-36 scores from participants with and without post-acute sequelae of COVID-19 (PASC).

SF-36 Scales	PASC N = 32	No PASC N = 30	*p*-Value
Physical Functioning	57.6 ± 30.4	92.7 ± 17.0	<0.0001
Role Limitations due to Physical Health	41.9 ± 38.3	75.3 ± 37.1	<0.001
Emotional Well Being	69.9 ± 23.7	78.5 ± 15.1	0.10
Role limitations due to Emotional Problems	58.2 ± 40.6	82.2 ± 28.7	<0.01
Energy/Fatigue	36.4 ± 21.4	63.8 ± 24.5	<0.0001
Pain	66.5 ± 28.9	83.3 ± 24.0	<0.05
Social Functioning	69.3 ± 27.1	86.6 ± 19.6	<0.01
General Health	52.9 ± 20.8	79.7 ± 19.3	<0.0001

Values presented mean ± standard deviation.

**Table 5:** Comparison of SF-36 scores between adults with and without pre-existing chronic conditions.

SF-36 Scales	Chronic Conditions N = 28	No Chronic Conditions N = 34	*p*-Value
Physical Functioning	59.8 ± 32.2	86.7 ± 22.7	<0.001
Role Limitations due to Physical Health	45.5 ± 42.5	68.4 ± 37.3	<0.05
Emotional Well Being	73.3 ± 23.4	74.7 ± 17.7	0.78
Role limitations due to Emotional Problems	63.1 ± 40.9	75.4 ± 33.2	0.20
Energy/Fatigue	39.6 ± 26.6	57.9 ± 24.0	<0.01
Pain	69.4 ± 31.8	78.9 ± 23.5	0.18
Social Functioning	72.3 ± 25.3	82.1 ± 24.5	0.13
General Health	56.6 ± 23.3	73.5 ± 22.2	<0.01

Values presented are mean ± standard deviation. Pre-existing chronic conditions were collected from hospital electronic records and self-reported at clinic visits.

## Data Availability

All data in this manuscript is available through REDCap. Contact the corresponding author for data.

## Supplementary Materials

The following are available online at https://www.mdpi.com/article/10.3390/ijerph182111048/s1, Figure S1: Study design, Figure S2: Comparison of scales of the SF-36 for those who were hospitalized and those who were not hospitalized due to COVID-19, Figure S3: Comparison of those with PASC and those without PASC due to COVID-19 and the scales of the SF-36, Table S1: Comparison of SF-36 Scores between Males and Females with COVID-19, Table S2: Distribution of SF-36 scores by ethnicity, Table S3: Distribution of SF-36 scores according to the number of days post-SARS-CoV-2 PCR positive diagnostic test results, Table S4: Comparison of SF-36 scores across body mass index categories of COVID-19 participants.
