# Supplementary material for: Quality of Life (QoL) Is Reduced in Those with Severe COVID-19 Disease, Post-Acute Sequelae of COVID-19, and Hospitalization in United States Adults from Northern Colorado
Source: Int J Environ Res Public Health. 2021 Oct 21;18(21):11048. doi: 10.3390/ijerph182111048 (PMC8582735; doi:10.3390/ijerph182111048)
Supplement: Supplementary file 1 [file ijerph-18-11048-s001.zip › ijerph-1340489-supplementary.pdf]

Supplemental Table S1 Comparison of SF-36 Scores between Males and Females with COVID-19

SF-36 Scales	Male<br>N = 24	Female<br>N = 38	P-value
Physical Functioning	75.7 ± 28.4	73.8 ±31.8	0.81
Role Limitations due to Physical Health	55.6 ± 38.4	59.6 ± 43.0	0.71
Emotional Well Being	75.3 ± 19.3	73.3 ± 21.1	0.70
Role limitations due to Emotional Problems	66.7 ± 41.7	71.8 ± 34.3	0.60
Energy/Fatigue	55.0 ± 26.0	46.3 ± 26.8	0.21
Pain	86.7 ± 19.0	67.0 ± 29.9	< 0.01
Social Functioning	78.6 ± 26.4	77.0 ± 24.6	0.81
General Health	69.2± 22.6	63.8 ± 25.0	0.40

Values presented mean ± standard deviation.

Supplemental Table S2 Distribution of SF-36 scores by ethnicity

SF-36 Scales	Hispanic<br>N = 9	Non-Hispanic<br>N = 53	P-value
Physical Functioning	66.9 ± 34.0	75.9 ± 29.8	0.42
Role Limitations due to Physical Health	52.8 ± 45.8	59.0 ± 40.6	0.68
Emotional Well Being	81.8 ± 12.2	72.8 ± 21.2	0.22
Role limitations due to Emotional Problems	51.8 ± 41.2	72.9 ± 35.9	0.12
Energy/Fatigue	48.9 ± 30.7	48.8± 26.2	0.92
Pain	76.7 ± 30.8	74.3 ± 27.5	0.81
Social Functioning	83.3 ± 20.7	76.57± 25.9	0.47
General Health	68.9 ± 19.0	65.4 ± 25.20	0.69

Values presented mean ± standard deviation.

Supplemental Table S3 Distribution of SF-36 scores according to the number of days post PCR diagnostic test results

SF-36 Scales	15-44<br>N = 11	45-89<br>N= 16	90-175<br>N= 17	175+<br>N = 17	P-value
Physical Functioning	73.6 ± 31.1	69.4 ± 31.8	76.4 ± 34.6	76.8 ± 26.0	0.90
Role Limitations due to Physical Health	36.4 ± 39.3	46.9 ± 43.7	70.0 ± 38.2	68.2 ± 37.2	0.08
Emotional Well Being	84.7 ± 14.1	71.3 ± 21.5	73.5 ± 24.4	69.9 ± 17.6	0.27
Role limitations due to Emotional Problems	54.6 ± 45.4	68.7 ± 33.3	83.9 ± 29.5	64.9 ± 39.9	0.21
Energy/Fatigue	51.4 ± 24.2	45.0 ± 29.6	57.1 ± 27.7	43.8 ± 24.0	0.46
Pain	83.0 ± 21.3	73.0 ± 27.8	80.7 ± 28.1	64.4 ± 30.5	0.25
Social Functioning	69.3 ± 25.2	77.3 ± 25.9	83.3 ± 29.0	77.3 ± 25.9	0.58
General Health	66.4 ± 18.7	64.4 ± 22.5	76.4 ± 23.6	64.4 ± 22.5	0.09

Values presented mean ± standard deviation.

Supplemental Table S4. Comparison of SF-36 scores across body mass index categories of COVID-19 participants

**SF-36 Scales**	**Normal or Under Weight N=20**	**Overweight N=15**	**Obese N= 27**	**P-value**
Physical Functioning	91.1 ± 16.5	71.7 ± 33.8	64.0 ± 31.8*	< 0.01
Role Limitations due to Physical Health	80.8 ± 31.9	50.7 ± 34.4	45.4 ± 44.4*	< 0.01
Emotional Well Being	79.1 ± 12.6	77.1 ± 12.8	68.7 ± 26.6	0.18
Role limitations due to Emotional Problems	86.3 ± 20.4	60.2 ± 42.2	62.9 ± 40.7	0.05
Energy/Fatigue	60.3 ± 25.8	53.3 ± 23.4	39.8 ± 26.2*	< 0.05
Pain	81.0 ± 21.3	85.0 ± 21.1	64.2 ± 32.0*	< 0.05
Social Functioning	87.0 ± 22.1	80.8 ± 22.1	69.0 ± 26.7*	< 0.05
General Health	78.9 ± 20.4	70.3 ± 23.0	53.7 ± 21.7*	< 0.001

*Significantly different from Mild and Normal or Underweight. Note: only three participants were under-weight by BMI categories, but all 3 were close to the cutoff. These categories did not take into account participant's build or diet. Values presented as mean ± standard deviation.
